# Imaging features of intracerebral hemorrhage with cerebral amyloid angiopathy: Systematic review and meta-analysis

**DOI:** 10.1371/journal.pone.0180923

**Published:** 2017-07-10

**Authors:** Neshika Samarasekera, Mark Alexander Rodrigues, Pheng Shiew Toh, Rustam Al-Shahi Salman

**Affiliations:** Centre for Clinical Brain Sciences, University of Edinburgh, Edinburgh, United Kingdom; University of Toronto, CANADA

## Abstract

**Background:**

We sought to summarize Computed Tomography (CT)/Magnetic Resonance Imaging (MRI) features of intracerebral hemorrhage (ICH) associated with cerebral amyloid angiopathy (CAA) in published observational radio-pathological studies.

**Methods:**

In November 2016, two authors searched OVID Medline (1946-), Embase (1974-) and relevant bibliographies for studies of imaging features of lobar or cerebellar ICH with pathologically proven CAA (“CAA-associated ICH”). Two authors assessed studies’ diagnostic test accuracy methodology and independently extracted data.

**Results:**

We identified 22 studies (21 cases series and one cross-sectional study with controls) of CT features in 297 adults, two cross-sectional studies of MRI features in 81 adults and one study which reported both CT and MRI features in 22 adults. Methods of CAA assessment varied, and rating of imaging features was not masked to pathology. The most frequently reported CT features of CAA-associated ICH in 21 case series were: subarachnoid extension (pooled proportion 82%, 95% CI 69–93%, I^2^ = 51%, 12 studies) and an irregular ICH border (64%, 95% CI 32–91%, I^2^ = 85%, five studies). CAA-associated ICH was more likely to be multiple on CT than non-CAA ICH in one cross-sectional study (CAA-associated ICH 7/41 vs. non-CAA ICH 0/42; χ^2^ = 7.8, p = 0.005). Superficial siderosis on MRI was present in 52% of CAA-associated ICH (95% CI 39–65%, I^2^ = 35%, 3 studies).

**Conclusions:**

Subarachnoid extension and an irregular ICH border are common imaging features of CAA-associated ICH, but methodologically rigorous diagnostic test accuracy studies are required to determine the sensitivity and specificity of these features.

## Introduction

About 85% of ‘primary’ intracerebral haemorrhages (ICHs) have no specific underlying cause and are attributed to cerebral small vessel diseases, mostly arteriolosclerosis or cerebral amyloid angiopathy (CAA).[1,2] Arteriolosclerosis may underlie ICH in any location, whereas CAA only affects cortical and leptomeningeal vessels and is therefore associated with lobar and cerebellar ICH.[3] Accurate diagnosis of lobar or cerebellar ICH accompanied by pathologically-proven CAA (“CAA-associated ICH”) is important given its higher risk of recurrent ICH, anticoagulant-associated ICH and post-stroke dementia compared with arteriolosclerosis-associated ICH.[2,4]

Whilst definitive diagnosis of CAA requires histopathology, tissue samples from living patients are rarely available. In contrast, brain imaging is commonly performed in ICH, and may help differentiate these underlying pathologies. The magnetic resonance imaging (MRI)-based modified Boston criteria attribute lobar or cerebellar ICH to ‘probable CAA’ without pathological confirmation if there is either at least one other lobar or cerebellar ICH on brain imaging, or if at least one lobar cerebral or cerebellar microbleed or cortical superficial siderosis is present on gradient-echo brain MRI sequences.[5,6] The criteria have excellent sensitivity (95%, 95% CI 83–99%) and good specificity (81%, 95% CI 62–93%) in a hospital-based cohort with ICH,[5] but have not been externally validated. Positron emission tomography (PET) can directly detect amyloid ante-mortem. Whilst it shows good sensitivity (92%, 95% CI 72–99%) for presumed CAA-associated lobar ICH, its specificity is limited (65%, 95% CI 50–78%), reflecting the high frequency of parenchymal amyloid plaques among the healthy elderly.[7] Another drawback of MRI- and PET-based diagnostic criteria for CAA-associated ICH is the limited access to advanced imaging; these techniques may not be suitable in the acute setting, nor available, particularly in middle and low-income countries, where 75% of deaths from haemorrhagic stroke now occur.[8] In contrast, computed tomography (CT) is widely available, has few contraindications and is frequently used as the first test to investigate stroke. Therefore determining whether any CT features of ICH and the rest of the brain discriminate between CAA-associated ICH and other ICH is important.

The primary objective of this study was to systematically review and meta-analyse published diagnostic test accuracy studies or other observational radio-pathological studies describing features of CAA-associated lobar or cerebellar ICH.

## Materials and methods

### Search strategy and selection criteria

In November 2016 two authors (NS and PST) searched Ovid Medline (1946-) and Embase (1974-) using comprehensive electronic search strategies (S1 Appendix). One author (PST) also searched the bibliographies of relevant publications and Google scholar for other papers citing each included paper. We also searched our personal files. We did not publish a review protocol. We performed the study according to the PRISMA statement (S1 Checklist).[9]

### Eligibility criteria

Published studies were eligible for inclusion if they described imaging features of lobar or cerebellar ICH associated with CAA, proven pathologically by brain biopsy, haematoma evacuation or post-mortem (primary outcome), regardless of sample size, language of publication, and study design.

### Data collection

Two authors (NS and PST) screened all titles and abstracts for eligibility, removed duplicates and read the full text of articles that were potentially eligible for inclusion. Eligible studies were read in full by two authors (NS and PST) who extracted data independently on: study design, methods of assessment and grading of CAA, participant characteristics and imaging features of ICH. We resolved disagreements by discussion. If pertinent study attributes or data were unavailable or unclear in an eligible publication, we sought clarification from the authors by email.

### Methodological assessment

Two authors (NS and PST) assessed each study using three items from the Joanna Briggs Critical Appraisal checklist for descriptive studies and case series^s1^ (sample selection, definition of inclusion criteria, and identification of confounding factors [in particular patient age and dementia]) in addition to assessing whether rating of imaging features was blinded to pathological findings and vice versa. For cross-sectional studies, we applied the QUADAS-2 tool which judges the quality of diagnostic test accuracy studies based upon patient selection, the index test, reference standard and participant flow through the study.^s2^

### Statistical analysis

If we identified multiple publications relating to the same cohort, we included only the largest study. For each case-series study, we determined the numbers of participants with lobar or cerebellar ICH accompanied by pathologically-proven CAA anywhere in the brain (“CAA-associated ICH”) and the frequency of the reported imaging characteristics of these participants’ brains. NS used the Stata command ‘metaprop’ to assess the prevalence of each imaging characteristic in CAA-associated ICH as a pooled proportion in a random-effects model with computation of confidence intervals using the ‘score’ method (Stata version 11.1, StataCorp LP, College Station, USA). NS assessed inconsistency using the I^2^ statistic. We sought to stratify our analyses by pre-ICH history of hypertension (present or not), first-ever vs. recurrent ICH and supratentorial lobar versus cerebellar ICH. We also assessed the frequency of reported combinations of imaging characteristics of CAA-associated ICH.

## Results

**Fig 1:**
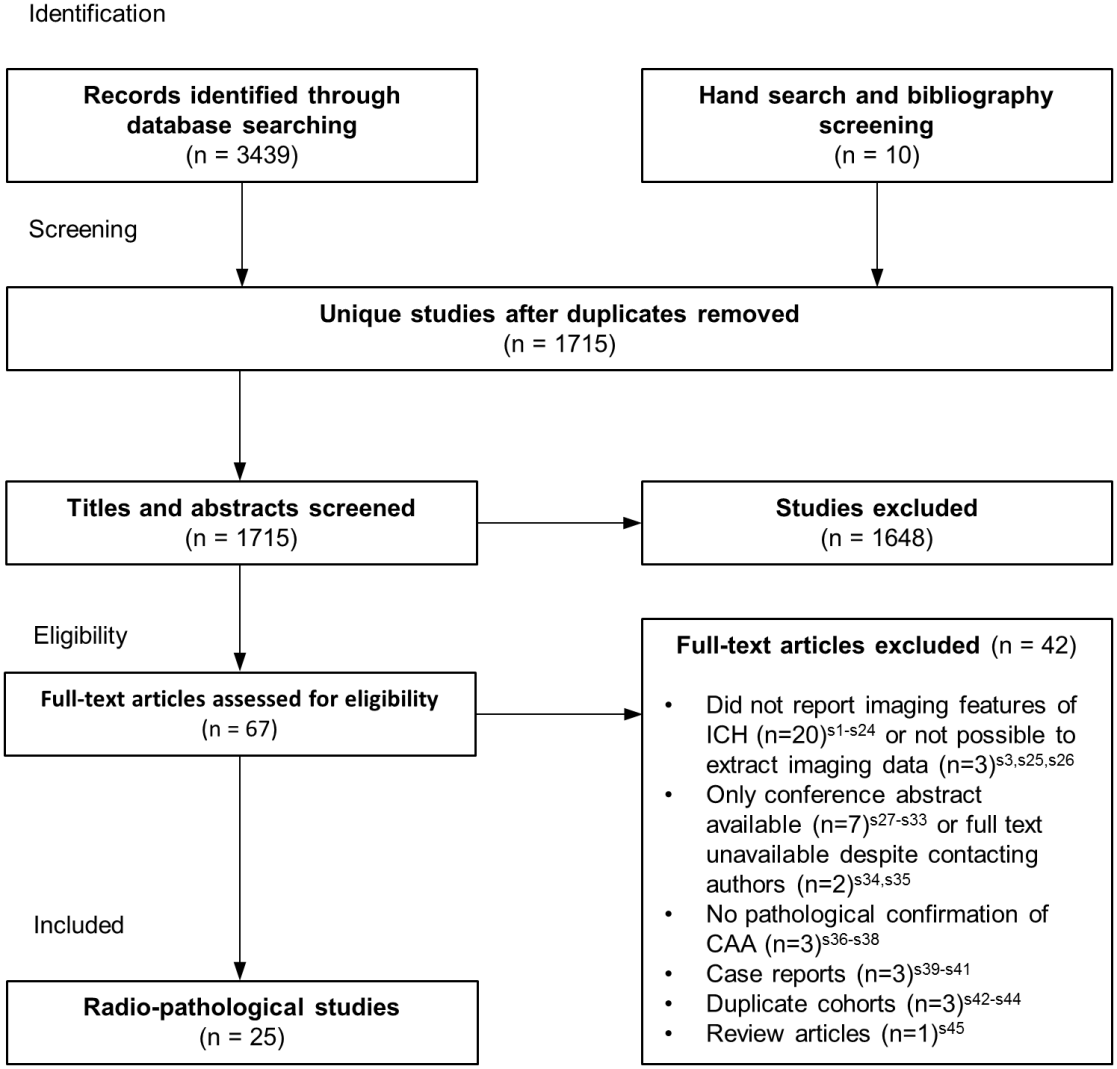
Flowchart of studies.

Our search strategies identified 1,715 articles of which 22 (one cross-sectional study with controls [10] and 21 case series without controls [11–31]) described CT features of CAA-associated lobar or cerebellar ICH in 297 adults, two studies described MRI features [5,32] in 81 adults and one cross-sectional study [33] described both CT and MRI features in 22 adults (Fig 1). There were no PET studies. We excluded one hospital-based cross-sectional study^s3^ which did not report ICH location per participant and whose applicability to this review raised concern because of the age (mean 58 years; standard deviation 11) and haematoma locations (185/421 [44%] in the basal ganglia) of those with CAA-associated ICH.

### Methodological quality of included studies

All 21 case series were hospital-based, using either consecutive cases in three (14%) studies [13,18,20] or selected hospital cases in the remainder, with a median sample size of 14 (IQR 7–37) (Table 1). Nine (43%) stated inclusion criteria.[11,13–15,17–21] Studies were retrospective bar one that selected consecutive patients from a prospectively recruited cohort.[13]

**Table 1:** Characteristics of included studies.

Study (Year)	Study Design	CT or MRI	Participants (n)	CAA-associated lobar or cerebellar ICH n (%)	Age range or mean age+/-SD (years)	First ever ICH n (%)	Cerebellar ICH n (%)	Pre-ICH hypertension, n (%)	Pre-ICH dementia, n (%)	Interval between symptom onset & diagnostic scan	Interval between diagnostic scan & pathological sampling	No. of raters of imaging characteristics per case
Doden (2016)[33]	Cross-sectional	Both	253	22 (9)	75+/-8	?	?	12 (55)	5 (23)	?	?	1
Hirohata (2010)[11]	Case-series	CT	303	41 (14)	55–85	?	1 (2)	?	6 (15)	?	?	?
Panicker (2010)[12]	Case-series	CT	3	3 (100)	65–78	?	0	1 (33)	0 (0)	?	?	?
Patel (2009)[13]	Case-series	CT	200	12 (6)	?	?	0	?	?	?	?	2
Chen (2004)[14]	Case-series	CT	5	5 (100)	65–83	?	0	1 (20)	0(0)	?	?	?
Oide (2003)[15]	Case-series	CT	64	64 (100)	61–91	42 (66)	1 (2)	19 (30)	15 (23)	?	?	?
Lang (2001)[10]	Cross-sectional	CT	83	41 (49)	53–90	?	2 (5)	?	?	?	?	5
Wang (2000)[16]	Case-series	CT	47	2 (4)	18–83	?	?	1 (50)	?	?	?	?
Izumihara (1999)[17]	Case-series	CT	37	37 (100)	61–91	26 (70)	0	15 (41)	7 (19)	?	?	?
Hagen (1999)[18]	Case-series	CT	14	13 (93)	64–79	?	0	6 (46)	?	?	?	?
Millar (1999)[19]	Case-series	CT	17	5 (29)	60–86	4(80)	0	5 (100)	1 (20)	?	1–54 days	?
Minakawa (1995)[20]	Case-series	CT	19	10 (53)	61–80	?	0	?	?	1–48 hours	0–2 days	?
Wakai (1992)[21]	Case-series	CT	29	6 (21)	65–76	5 (83)	0	2 (33)	?	?	0-≥15 days	?
Yong (1992)[22]	Case-series	CT	6	6 (100)	54–86	?	1 (17)	1 (17)	2 (33)	0-≥2 weeks	?	?
Xu (1990)[23]	Case-series	CT	11	11 (100)	45–79	?	0	5 (46)	?	0–5 days	?	?
Andoh (1989)[24]	Case-series	CT	37	3 (8)	67–73	3 (100)	0	1 (33)	?	?	0–14 days	?
Sobel (1985)[25]	Case-series	CT	2	2 (100)	67–72	1 (50)	0	1 (50)	0 (0)	?	?	?
Cosgrove (1985)[26]	Case-series	CT	24	6 (25)	58–88	?	0	#	?	?	?	?
Brown (1985)[27]	Case-series	CT	12	12 (100)	62–89	7 (58)	0	6 (50)	1 (8)	?	?	?
Gray (1985)[28]	Case-series	CT	12	3 (25)	55–83	?	0	1 (33)	3 (100)	?	?	?
Kalyan Raman (1984)[29]	Case-series	CT	10	6 (60)	54–88	?	1 (17)	1 (17)	0 (0)	?	48 hours-1 month	?
Patel (1984)[30]	Case-series	CT	2	2 (100)	67–77	0 (0)	0	?	1 (50)	?	?	?
Wagle (1984)[31]	Case-series	CT	7	7 (100)	62–84	6 (86)	0	3 (43)	2 (29)	0–5 weeks	?	?
Charidimou (2015)[32]	Cross-sectional	MRI	105	54 (51)	69–74	?	0	30 (58)	?	Median 1.3 months	?	?
Linn (2010)[5]	Cross-sectional	MRI	60	27 (45)	?	?	?	?	?	?	?	2

CAA = cerebral amyloid angiopathy, CT = computed tomography, ICH = intracerebral haemorrhage, MRI = magnetic resonance imaging.

^?^ = not reported,

^#^ = characteristic present in cohort but frequency unspecified

Of the two retrospective hospital-based cross-sectional studies using CT,[10,33] one included 41 cases [10] and the other 22 cases.[33] Both had a high risk of bias owing to inclusion of selected cases, potential misclassification since not all participants received the same reference standard and an unknown time interval between the index test (brain imaging) and the reference standard (histopathological diagnosis). The two cross-sectional studies assessing solely MRI were both retrospective hospital-based studies.[5,32] Again there was risk of bias due to potential misclassification since not all participants received the same reference standard.

Nine (36%) studies [15,17,19,21,24,25,27,30,31] distinguished first-ever from recurrent ICH. A pre-ICH history of hypertension or dementia were described in 19 (76%) [12,14–19,21–29,31–33] and 14 (56%) studies [11,12,14,15,17,19,22,25,27–31] respectively. Only four (16%) studies stated the interval between symptom onset and the diagnostic scan [20,22,23,31] and six (24%) studies stated the interval between the diagnostic scan and post-mortem or biopsy.[19–21,24,29,32] The number of raters of radiological features was mentioned in only four (16%) studies,[5,10,13,33] three of which used at least two raters per case.[5,10,13] Disagreements were resolved by consensus in two of these,[5,13] whilst the other study did not specify this.[10]

#### Pathological assessment

The extent of brain sampling for CAA varied considerably between the studies (Table 2). All studies bar two (which did not state how the authors ascertained CAA [5,19]) used Congo Red staining, nine of which also used immunohistochemistry for all [15,17,22] or some cases.[11,13,21,24,32,33] Two studies described the severity of CAA rather than its presence or absence.[12,26] None of the studies stated whether CAA was rated blind to imaging findings or whether more than one rater rated CAA.

**Table 2:** Pathology assessment in included studies.

Study (Year)	Number of tissue blocks examined	CAA sampling: Post-mortem/biopsy/ICH evacuation	Locations examined in cases	CAA detection	Rating of severity of CAA	Number of raters of CAA
Doden (2016)[33]	?	0/?/?	Haematoma site	CR &IHC	Presence/Absence	?
Hirohata (2010)[11]	?	9/32/0	?	CR & IHC^a^	Presence/Absence	?
Panicker (2010)[12]	?	1/2/0	?	CR	Vonsattel^e4^	?
Patel (2009)[13]	?	2/10/0	Haematoma site, L	CR or IHC	Presence/Absence	1
Chen (2004)[14]	?	0/5/0	Haematoma site	CR	Presence/Absence	?
Oide (2003)[15]	1 or 2	12/52/0	L	CR & IHC	Presence/Absence	?
Lang (2001)[10]	1	0/41/0	Haematoma site	CR	Presence/Absence	?
Wang (2000)[16]	?	0/2/0	Haematoma site	CR	Presence/Absence	?
Izumihara (1999)[17]	?	0/37/0	Haematoma site	CR & IHC	Presence/Absence	?
Hagen (1999)[18]	?	0/13/0	Haematoma site	CR	Presence/Absence	?
Millar (1999)[19]	?	5/0/0	?	?	Presence/Absence	?
Minakawa (1995)[20]	?	0/10/0	Haematoma site	CR	Presence/Absence	?
Wakai (1992)[21]	1	0/6/0	Haematoma site	CR & if +ve IHC	Presence/Absence	?
Yong (1992)[22]	?	0/6/0	Haematoma site	CR & IHC	Presence/Absence	?
Xu (1990)[23]	?	11/0/0	Haematoma site	CR	Presence/Absence	?
Andoh (1989)[24]	?	0/3/0	Haematoma site	CR or IHC	Presence/Absence	?
Sobel (1985)[25]	?	2/0/0	?	CR	Presence/Absence	?
Cosgrove (1985)[26]	≥5	6/0/0	L, HC, BG, M, C	CR	Author’s own	1
Brown (1985)[27]	?	7/5/0	?	CR	Presence/Absence	?
Gray (1985)[28]	3	3/0/0	F, P-O,BG	CR	Presence/Absence	?
Kalyan Raman (1984)[29]	11	4/2/0	F, T, P, O, C, HC, BG, M,P, Me, Haematoma	CR, Thioflavin T	Presence/Absence	?
Patel (1984)[30]	1	0/2/0	?	CR	Presence/Absence	?
Wagle (1984)[31]	?	5/2/0	?	CR	Presence/Absence	?
Charidimou (2015)[32]	?	?/?/?	?	CR or IHC	Vonsattel^e4^	?
Linn (2010)[5]	?	?/?/?	?	?	?	?

Locations: F = frontal lobe, P = parietal lobe, O = occipital lobe, P-O = Parieto-occipital region, T = temporal lobe, L = lobar unspecified, C = cerebellum, CC = corpus callosum, BG = basal ganglia, HC = hippocampus, M = midbrain, Me = medulla, Haematoma site = haematoma itself and/or adjacent parenchyma. CAA = cerebral amyloid angiopathy, CR = Congo red stain, ICH = intracerebral haemorrhage, IHC = immunohistochemistry,

^?^ = not reported,

^a^-IHC in 23

### CT features of CAA-associated ICH

#### Characteristics of included participants in CT studies

The majority of ICHs in the 319 participants were supratentorial lobar (n = 313). Three had a cerebellar ICH, one ICH was supratentorial lobar and cerebellum and two participants had a cerebellar ICH (but we could not determine whether it had occurred in isolation).

#### Frequency of CT features of CAA-associated ICH

**Fig 2:**
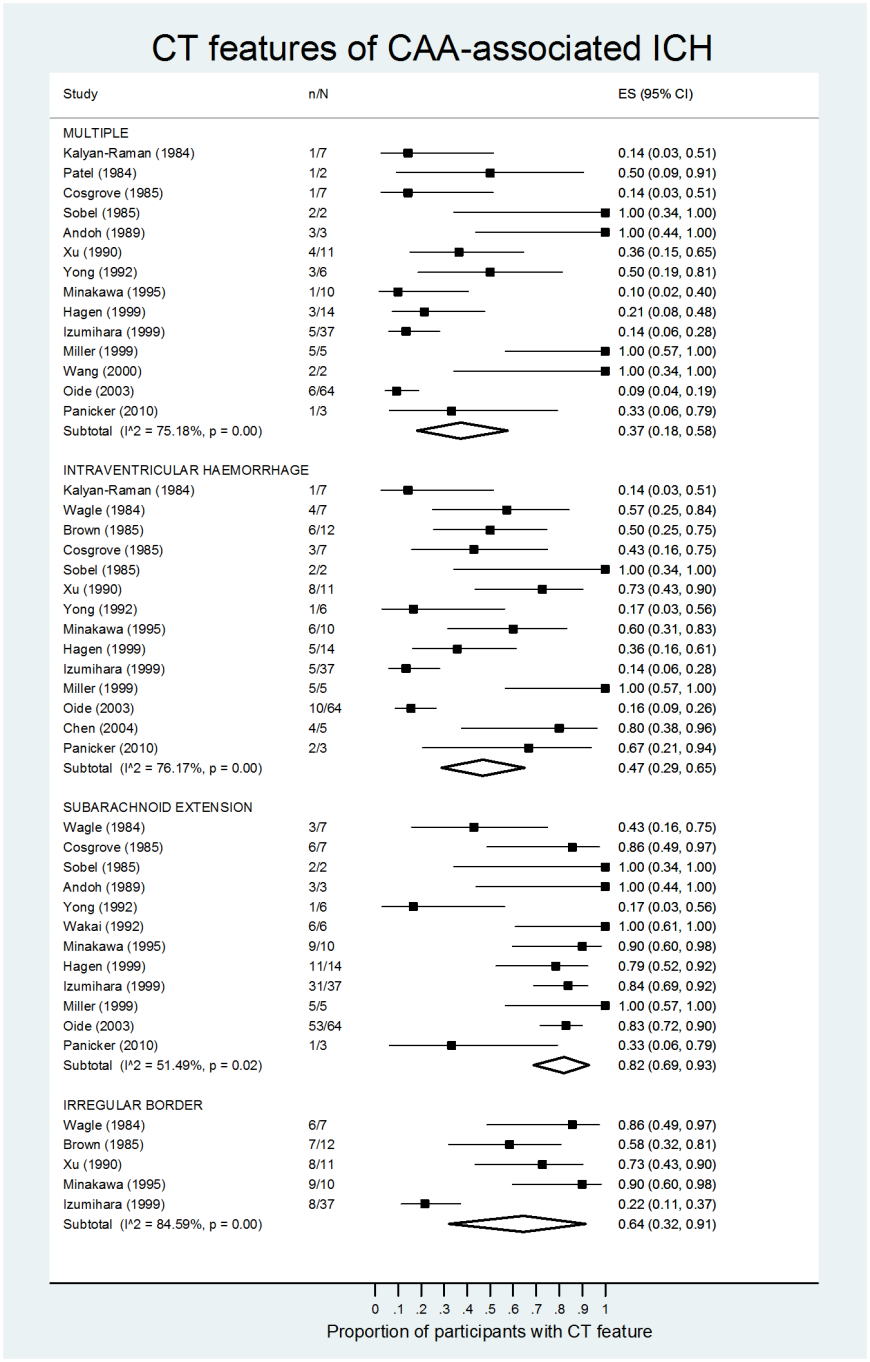
CT features of CAA-associated ICH–Pooled proportion meta-analysis, stratified by imaging feature. ES = effect size with 95% confidence intervals, n = number of participants with feature, N = number of participants with CAA-associated ICH.

The most frequently reported imaging features of CAA-associated ICH in case series without controls were: multiple simultaneous ICHs (19 studies), intraventricular extension of ICH (15 studies), subarachnoid extension (14 studies) and an irregular ICH border (6 studies) (Table 3). The pooled proportion of participants with subarachnoid extension was 82% (95% CI 69–93%, I^2^ = 51%, 12 studies), irregular ICH border 64% (95% CI 32–91%, I^2^ = 85%, 5 studies), intraventricular hemorrhage 47% (95% CI 29%-65%, I^2^ = 76%, 14 studies) and multiple ICHs 37% (95% CI 18–58%, I^2^ = 75%, 14 studies), (Fig 2 and Table 3).

**Table 3:** Imaging characteristics in CAA-associated ICH.

Study (Year)	CT or MRI	Sample Size	Multiple ICH, n (%)	Intraventricular extension, n (%)	Subarachnoid extension, n (%)	Subdural extension, n (%)	Blood\fluid level in a haematoma, n (%)	Variable density in a haematoma, n (%)	Superficial location, n (%)	Irregular border, n (%)	Lobulated ICH, n (%)	Surrounding oedema, n (%)	Periventricular WM lucency, n (%)	Superficial siderosis, n (%)	Lobar BMBs, n (%)	Severe WMH, n (%)
Doden (2016)[33]	Both	22		14 (64)	11 (50)	?	?	?	?	?	?	?	?	6 (35)*	#	#
Hirohata (2010)[11]	CT	41	#	#	#	?	?	?	?	?	?	?	?	?	?	?
Panicker (2010)[12]	CT	3	1 (33)	2 (67)	1 (33)	?	?	?	?	?	?	3 (100)	?	?	?	?
Patel (2009)[13]	CT	12	?	?	?	8 (67)	?	?	?	?	?	?	?	?	?	?
Chen (2004)[14]	CT	5	0 (0)	4 (80)	?	?	?	?	?	?	?	?	?	?	?	?
Oide (2003)[15]	CT	64	6 (9)	10 (16)	53 (83)	?	?	?	?	?	?	?	27 (42)	?	?	?
Lang (2001)[10]	CT	41	7 (17)	10 (24)	26 (63)	?	?	?	?	15 (37)	17 (41)	?	?	?	?	?
Wang (2000)[16]	CT	2	2 (100)	?	?	?	?	?	?	?	?	?	?	?	?	?
Izumihara (1999)[17]	CT	37	5 (14)	5 (14)	31 (84)	9 (24)	?	?	?	8 (22)	23 (62)	?	?	?	?	?
Hagen (1999)[18]	CT	13	3 (23)	5 (38)	11 (85)	2 (15)	?	?	?	?	?	?	?	?	?	?
Millar (1999)[19]	CT	5	5 (100)	5 (100)	5 (100)	?	3 (60)	5 (100)	5 (100)	#	5 (100)	?	5 (100)	?	?	?
Minakawa (1995)[20]	CT	10	1 (10)	6 (60)	9 (90)	?	7 (70)	?	?	9 (90)	?	?	?	?	?	?
Wakai (1992)[21]	CT	6	0 (0)	?	6 (100)	?	?	?	?	?	6 (100)	?	?	?	?	?
Yong (1992)[22]	CT	6	3 (50)	1 (17)	1 (17)	1 (17)	?	1 (17)	?	?	?	1 (17)	?	?	?	?
Xu (1990)[23]	CT	11	4 (36)	8 (73)	?	?	8 (73)	?	?	8 (73)	?	8 (73)	?	?	?	?
Andoh (1989)[24]	CT	3	3 (100)	?	3 (100)	?	?	1 (33)	1 (33)	?	3 (100)	?	?	?	?	?
Sobel (1985)[25]	CT	2	2 (100)	2 (100)	2 (100)	?	?	?	?	?	?	?	?	?	?	?
Cosgrove (1985)[26]	CT	6	1 (17)	3 (50)	6 (100)	3 (50)	?	?	?	?	?	?	?	?	?	?
Brown (1985)[27]	CT	12	0 (0)	6 (50)	#	?	?	?	?	7 (58)	?	?	?	?	?	?
Gray (1985)[28]	CT	3	?	?	?	?	?	?	?	?	?	?	3 (100)	?	?	?
Kalyan Raman (1984)[29]	CT	6	1 (17)	1 (17)	?	1 (17)	?	?	?	?	?	?	?	?	?	?
Patel (1984)[30]	CT	2	1 (50)	?	?	?	?	?	?	?	?	?	?	?	?	?
Wagle (1984)[31]	CT	7	0 (0)	4 (57)	3 (43)	?	?	?	3 (43)	6 (86)	?	6 (86)	?	?	?	?
Charidimou (2015)[32]	MRI	54	?	?	?	?	?	?	?	?	?	?	?	28 (52)	36 (67)	13 (24)
Linn (2010)[5]	MRI	27	?	?	?	?	?	?	?	?	?	?	?	17 (63)	?	?

^#^ = characteristic observed with CAA-associated ICH but frequency not stated.

BMB = brain microbleeds, CT = computed tomography, ICH = Intracerebral haemorrhage, MRI = magnetic resonance imaging, WM = white matter, WMH = white matter hyperintensities,

^?^ = not reported,

* MRI performed in 17 participants

17 studies described the locations of 346 ICHs in 262 patients.[10–12,14,15,17,18,20,22–27,29–31] The most frequent ICH locations were the frontal (83 [24%]) and parietal (83 [24%]) lobes. 72 (21%) ICHs were occipital, 58 (17%) were located in the temporal lobes and 7 (2%) were cerebellar. The remaining 43 ICHs involved multiple supratentorial lobes, with the parieto-occipital (15 [4%]) and fronto-parietal (14 [4%]) regions being the most frequently involved.

In one cross-sectional study,[10] CAA-associated ICHs were more likely to be multiple (CAA-associated ICH 7/41 vs. non-CAA ICH 0/42; χ^2^ = 7.8, p = 0.005) and have subarachnoid haemorrhage (CAA-associated ICH 26/41 vs. non-CAA ICH 11/42; p<0.05) compared to non-CAA ICHs. CAA-associated ICHs were also more likely be lobulated in comparison to non-CAA ICH (CAA-associated ICH 17/41 vs. non-CAA ICH 5/42; χ^2^ = 9.3, p = 0.002) but the proportion of ICHs with an irregular border did not differ between groups (CAA-associated ICH 15/41 vs. non-CAA ICH 22/42; χ^2^ = 2.1, p>0.05). However, the study did not explicitly define the terms ‘lobulated’ or ‘irregular border’. There was no difference in intraventricular haemorrhage between groups (CAA-associated ICH 10/41 vs. non-CAA ICH 11/42; p>0.05). In another cross-sectional study [33] there was no difference in either intraventricular hemorrhage (CAA-associated ICH 14/22 vs. non-CAA ICH 4/9; p = 0.279) or focal subarachnoid hemorrhage (CAA-associated ICH 11/22 vs. non-CAA ICH 2/9; p = 0.154) between groups.

CT features of CAA-associated ICH did not differ in first-ever vs recurrent ICH (S1 Fig) or those with a history of pre-stroke hypertension vs. those without (S2 Fig). We were unable to compare imaging features in lobar vs. cerebellar ICH given the paucity of infratentorial ICHs (n = 6). In 11 studies [12,18–22,25,27–29,31] of 75 ICHs reporting imaging features for each case of CAA-associated ICH, the three most frequently paired features were: intraventricular and subarachnoid extension (n = 21 [28%]), an irregular ICH border with subarachnoid extension (n = 14 [19%]) and a blood or fluid level within the haematoma with subarachnoid extension (n = 12 [16%]).

### MRI features of CAA-associated ICH

#### Characteristics of included studies and participants

We identified three retrospective, hospital-based cross-sectional studies, one of CAA-associated ICH which fulfilled the Boston criteria for CAA (we assumed CAA-associated ICH was either lobar or cerebellar [n = 27] vs. non-CAA ICH [n = 22]),[5] one of CAA-associated lobar ICH (n = 54) vs CAA without ICH (n = 51) [32] and a cross sectional study which used MRI on a subset of 17 adults with cortico-subcortical (i.e. lobar) ICH which fulfilled the Boston criteria for CAA vs. non-CAA ICH (n = 7).[33]

#### Frequency of MRI features of CAA-associated ICH

The pooled proportion of focal or disseminated superficial siderosis in CAA-associated ICH was 52% (95% CI 39–65%, I^2^ = 35%, 3 studies; S3 Fig). The imaging features of ICH reported in one study were lobar brain microbleeds (n = 36 [67%]), severe centrum semi-ovale enlarged perivascular spaces (n = 29 [55%]), severe white matter hyperintensities (n = 13 [24%]) and severe basal ganglia enlarged perivascular spaces (n = 11 [21%]).[32] We excluded one study from this analysis which used CT to report white matter hyperintensities and did not report the frequency of lobar brain microbleeds.[33]

None of the studies assessing MRI features of CAA-associated ICH described ICH locations.

#### Sensitivity and specificity of the Boston criteria for detection of CAA-associated ICH

From one study (n = 31) the sensitivity of the Boston criteria was 32% (95% CI 16–53%) and the specificity was 78% (95% CI 45–94%), however 7/31 participants did not have MRI.[33]

## Discussion

In this systematic review and meta-analysis of 25 radio-pathological studies of 400 adults with CAA-associated lobar or cerebellar ICH, the most common CT imaging features in CAA-associated ICH were extension of the ICH into the subarachnoid space and an irregular ICH border. We found no difference in these CT imaging features of CAA-associated ICH in people with vs. without a pre-stroke history of hypertension and people with first-ever vs. recurrent lobar ICH. Superficial siderosis on MRI was visible in more than half of those with CAA-associated ICH.

This review benefited from comprehensive search strategies to ascertain relevant studies and thorough critical appraisal of all identified studies by two independent authors. However, study quality was limited, since most studies were hospital-based case series, which did not report the interval between symptom onset and the diagnostic scan (which may affect the imaging appearances of an ICH), had no blinding of assessors and lacked definitions of imaging variables or CAA-associated ICH.

We demonstrated that subarachnoid extension was a frequent finding in CAA-associated ICH, which may be in keeping with CAA preferentially affecting leptomeningeal and cortical vessels which could rupture into the subarachnoid space.[34] However, this may also be due to the selection bias of pathological studies, since subarachnoid extension is associated with larger ICH volumes,[35] or simply anatomy rather than underlying pathology since subarachnoid extension is more frequent in lobar ICH compared to non-lobar ICH.[1]

CAA-associated ICHs frequently had an irregular border. Whilst the pathophysiology of this finding is not fully understood, it may reflect deficiencies in endothelial and subendothelial functioning in CAA-affected vessels and/or impaired vasoconstriction, resulting in detrimental effects on haemostasis.[10,36,37] Alternatively, selection bias in radio-pathological studies may account for this finding since an irregular ICH border is associated with death.[38]

Although we found no difference in imaging characteristics between people with first-ever and recurrent ICH, the sample size was small. Multiple ICH was more frequent in CAA-associated ICH in one cross-sectional study, and both multiple and recurrent ICH may be in keeping with a biological gradient for CAA that is yet to be established.[3] Similarly we could not detect a difference between people with pre-ICH hypertension and those without, although this may be due to selection bias, variable definitions of hypertension, inclusion of people with both first-ever and recurrent ICH, or small sample sizes.

Nearly half of people with CAA-associated ICH in MRI studies did not have superficial siderosis or severe centrum semiovale enlarged perivascular spaces and nearly one third did not have lobar microbleeds, underlining the need for further studies to determine which MRI features–alone or in combination–are most sensitive for CAA-associated ICH. The modified Boston criteria had limited sensitivity with reasonable specificity in a small hospital-based cohort of adults with ICH, although not all participants underwent MRI.[33] Nonetheless, this highlights the requirement for external validation of these criteria to better define their diagnostic accuracy.

Differentiating CAA- and non-CAA-associated ICH is important because of potential differences in management and prognosis.[2,4] The optimal approach would use an imaging modality that is available worldwide, given that the greatest global burden of ICH is now in middle and low income countries.[8] CT, in contrast to MRI and PET, is widely available, has few contraindications and is frequently used in the acute setting for ICH. CT-based criteria for CAA-associated lobar ICH may therefore be more widely applicable and practical than those requiring advanced imaging such as MRI or PET. Our systematic review showed that no such criteria exist.

Future work should focus on performing well-designed studies to produce accurate diagnostic criteria for CAA-associated ICH with low risk of bias. The ideal study design would be a diagnostic test accuracy study of an unselected, population-based cohort, with a systematic examination of a pathological reference standard, examining the diagnostic utility of relevant imaging features, such as subarachnoid and intraventricular extension, irregular ICH border and multiple simultaneous acute ICHs identified in this review. The imaging features should clearly and reliably defined, particularly those that are subjective, such as ‘irregular’ or ‘lobulated’ ICH.[39,40] Given the variable assessment of CAA, its patchy distribution with vulnerability to sampling variation[41] and co-existence with other small vessel diseases,[42] a standard definition of ‘CAA-associated ICH’ is also essential. Pathological and imaging assessment should be blinded, and assessment by multiple raters is required to determine inter-observer variability.

In conclusion, this systematic review of radio-pathological studies showed that subarachnoid extension and an irregular border are the most common imaging features of CAA-associated lobar and cerebellar ICH. However, the diagnostic value of these and other imaging features needs to be assessed in rigorous diagnostic test accuracy studies. CT-based diagnostic criteria for CAA-associated ICH would be the most widely applicable, particularly for frail patients who cannot tolerate MRI and PET, and in low-middle income countries where advanced imaging is not available and the greatest global burden of ICH lies now and in the future.[8]

## Supporting information

S1 Appendix OVID Medline and Embase search strategies and supporting material references. (DOCX)

S1 Checklist PRISMA 2009 checklist. (DOC)

S1 Fig Meta-analysis of the relative risk of imaging features in first-ever vs. recurrent CAA-associated ICH. n = number of participants with feature, N = denominator, RR = relative risk, (95% CI) = 95% confidence intervals.
(TIF)

S2 Fig Meta-analysis of the relative risk of imaging features in people with CAA-associated ICH and a history of hypertension vs. people without a history of hypertension. n = number of participants with feature, N = denominator, RR = relative risk, (95% CI) = 95% confidence intervals.
(TIF)

S3 Fig Superficial siderosis in CAA-associated ICH—Pooled proportion meta-analysis. ES = effect size, n = number of participants with superficial siderosis, N = total number with CAA-associated ICH, (95% CI) = 95% confidence intervals.
(TIF)
