# Correction: Imaging features of intracerebral hemorrhage with cerebral amyloid angiopathy: Systematic review and meta-analysis

**DOI:** 10.1371/journal.pone.0187386

**Published:** 2017-10-27

**Authors:** Neshika Samarasekera, Mark Alexander Rodrigues, Pheng Shiew Toh, Rustam Al-Shahi Salman

The fourth author’s name is incorrect in the citation. The correct citation is: Samarasekera N, Rodrigues MA, Toh PS, Al-Shahi Salman R (2017) Imaging features of intracerebral hemorrhage with cerebral amyloid angiopathy: Systematic review and meta-analysis. PLoS ONE12(7): e0180923. https://doi.org/10.1371/journal.pone.0180923
