# Supplementary material for: Imaging features of intracerebral hemorrhage with cerebral amyloid angiopathy: Systematic review and meta-analysis
Source: PLoS One. 2017 Jul 10;12(7):e0180923. doi: 10.1371/journal.pone.0180923 (PMC5507310; doi:10.1371/journal.pone.0180923)
Supplement: S1 Appendix — (DOCX) [file pone.0180923.s001.docx]

# Supporting material

**S1 Appendix. OVID Medline search strategy**

1. Stroke/

2. Cerebrovascular Disorders/

3. exp basal ganglia cerebrovascular disease/ or exp brain ischemia/ or carotid artery diseases/ or carotid artery thrombosis/ or carotid stenosis/ or exp intracranial arterial diseases/ or exp "intracranial embolism and thrombosis"/ or exp intracranial hemorrhages/ or exp brain infarction/ or hypoxia-ischemia, brain/

4. ((brain$ or cerebr$ or cerebell$ or cortical or vertebrobasil$ or hemispher$ or intracran$ or intracerebr$ or infratentorial or supratentorial or mca$ or middle cerebr$ or anterior circulation or posterior circulation or basal ganglia or parenchyma$ or brain?stem or posterior fossa or ganglion$ or thalam$ or cortical) adj5 (isch?emi$ or infarct$ or thrombo$ or emboli$ or occlus$ or hypox$ or obstruction or vasculopathy)).tw.

5. ((lacunar or cortical) adj5 infarct$).tw.

6. ((brain$ or cerebr$ or cerebell$ or intracerebr$ or intracran$ or parenchyma$ or intraventricular or infratentorial or supratentorial or basal gang$ or ganglion$ or putaminal or putamen or posterior fossa or brain?stem or intra?axial or lobar or deep or thalam$ or cortical or superficial or vertebrobasil$ or front$ or tempor$ or pariet$ or occipit$) adj5 (haemorrhage$ or hemorrhage$ or haematoma$ or hematoma$ or bleed$)).tw.

7. ((h?emorrhag$ or isch?emi$) adj6 (stroke$ or cerebrovasc$ or cerebr?vasc$ or cerebral vasc$ or brain vasc$ or cva$ or apoplex$ or attack$ or event$ or insult$)).tw.

8. 1 or 2 or 3 or 4 or 5 or 6 or 7

9. exp Pathology, Clinical/

10. exp Amyloid beta-Protein/ or exp Amyloid/ or exp Amyloid beta-Protein Precursor/

11. exp Cerebral Amyloid Angiopathy/

12. exp Congo Red/

13. (cerebral amyloid angiopathy or congophil$ or congo?red or amyloid$ or A?beta or beta?amyloid).tw.

14. 10 or 11 or 12 or 13

15. (patholog$ or post?mortem$ or autops$ or necrops$ or biops$ or tissue$ or histo?patholog$ or neuro?patholog$ or clinic?patholog$).tw.

16. 9 or 15

17. 8 and 14 and 16

18. limit 17 to humans

**S2 Appendix. Ovid Embase search strategy**

1. cerebrovascular disease/

2. basal ganglion hemorrhage/ or cerebral artery disease/ or cerebrovascular accident/ or stroke/ or vertebrobasilar insufficiency/ or exp carotid artery disease/ or exp brain hematoma/ or exp brain hemorrhage/ or brain infarction/ or brain infarction size/ or brain stem infarction/or cerebellum infarction/ or exp brain ischemia/ or exp occlusive cerebrovascular disease/ or cerebellum injury/ or exp carotid artery/

3. ((h?emorrhag$ or isch?emi$) adj6 (stroke$ or cerebrovasc$ or cerebr?vasc$ or cerebral vasc$ or brain vasc$ or cva$ or apoplex$ or attack$ or event$ or insult$)).tw.

4. ((brain$ or cerebr$ or cerebell$ or cortical or vertebrobasil$ or hemispher$ or intracran$ or intracerebr$ or infratentorial or supratentorial or mca$ or middle cerebr$ or anterior circulation or posterior circulation or basal ganglia or parenchyma$ or brain?stem or posterior fossa or ganglion$ or thalam$ or cortical) adj5 (isch?emi$ or infarct$ or thrombo$ or emboli$ or occlus$ or hypox$ or obstruction or vasculopathy)).tw.

5. ((lacunar or cortical) adj5 infarct$).tw.

6. ((brain or cerebr$ or cerebell$ or intracerebr$ or intracran$ or parenchyma$ or intraventricular or infratentorial or supratentorial or basal gang$ or ganglion$ or putaminal or putamen or posterior fossa or brain?stem or intra?axial or lobar or deep or thalam$ or cortical or superficial or vertebrobasil$ or front$ or tempor$ or pariet$ or occipit$) adj5 (haemorrhage$ or hemorrhage$ or haematoma$ or hematoma$ or bleed$)).tw.

7. 1 or 2 or 3 or 4 or 5 or 6

8. "amyloid beta protein[1-42]"/ or exp amyloid/ or "amyloid beta protein[1-40]"/ or exp amyloid precursor protein/ or exp amyloid beta protein/

9. exp vascular amyloidosis/

10. exp congo red/

11. (cerebral amyloid angiopathy or congophil$ or congo?red or amyloid$ or A?beta or beta?amyloid).tw.

12. 8 or 9 or 10 or 11

13. exp pathology/

14. (patholog$ or post?mortem$ or autops$ or necrops$ or biops$ or tissue$ or histo?patholog$ or neuro?patholog$ or clinic?patholog$).tw.

15. 13 or 14

16. 7 and 12 and 15

17. limit 16 to human

## Supporting material references

s1 Joanna Briggs Institute. Joanna Briggs Critical Appraisal Checklist for Descriptive/Case series. 2015. 20-9-2015.

Ref Type: Online Source

s2 Whiting PF, Rutjes AW, Westwood ME, Mallett S, Deeks JJ, Reitsma JB et al. QUADAS-2: a revised tool for the quality assessment of diagnostic accuracy studies. *Ann Intern Med* 2011; 155(8):529-536.

s3 Tang YJ, Li Y, Wang S, Zhu MW, Sun YL, Zhao JZ. The incidence of cerebral amyloid angiopathy in surgically treated intracranial hemorrhage in the Chinese population. *Neurosurg Rev* 2013; 36(4):533-539.

s4 Vonsattel JP, Myers RH, Hedley-Whyte ET, Ropper AH, Bird ED, Richardson EP, Jr. Cerebral amyloid angiopathy without and with cerebral hemorrhages: a comparative histological study. *Annals of Neurology* 1991; 30(5):637-649.

s5Johnson KA, Gregas M, Becker JA, Kinnecom C, Salat DH, Moran EK et al. Imaging of amyloid burden and distribution in cerebral amyloid angiopathy. *Ann Neurol* 2007; 62(3):229-234.

s6 Mendel T, Wierzba-Bobrowicz T, Stepien T, Szpak GM. beta-amyloid deposits in veins in patients with cerebral amyloid angiopathy and intracerebral haemorrhage. *Folia Neuropathol* 2013; 51(2):120-126.

s7 Mendel TA, Wierzba-Bobrowicz T, Stepien T, Szpak GM. The association between cerebral amyloid angiopathy and atherosclerosis in patients with intracerebral hemorrhages. *Folia Neuropathol* 2013; 51(3):243-249.

s8 Holling M, Jeibmann A, Fischer BR, Albert FK, Ebel H, Paulus W et al. Histopathological analysis of intracerebral hemorrhage: implications for clinical management. *Acta Neurochir (Wien )* 2012; 154(3):439-443.

s9 De Reuck J, Auger F, Cordonnier C, Deramecourt V, Durieux N, Pasquier F et al. Comparison of 7.0-T T(2)*-magnetic resonance imaging of cerebral bleeds in post-mortem brain sections of Alzheimer patients with their neuropathological correlates. *Cerebrovasc Dis* 2011; 31(5):511-517.

s10 Xu D, Yang CH, Wang LN. [Prevalence and characteristics of cerebral amyloid angiopathy in the elderly]. *Zhonghua Nei Ke Za Zhi* 2003; 42(8):541-544.

s11 Izumihara A, Ishihara T, Hoshii Y, Ito H. Cerebral amyloid angiopathy associated with hemorrhage: immunohistochemical study of 41 biopsy cases. *Neurol Med Chir (Tokyo)* 2001; 41(10):471-477.

s12 Abrahams NA, Prayson RA. The role of histopathologic examination of intracranial blood clots removed for hemorrhage of unknown etiology: a clinical pathologic analysis of 31 cases. *Ann Diagn Pathol* 2000; 4(6):361-366.

s13 Fazekas F, Kleinert R, Roob G, Kleinert G, Kapeller P, Schmidt R et al. Histopathologic Analysis of Foci of Signal Loss on Gradient-Echo T2*-Weighted MR Images in Patients with Spontaneous Intracerebral Hemorrhage: Evidence of Microangiopathy-Related Microbleeds. *American Journal of Neuroradiology* 1999; 20(4):637-642.

s14 Reith W. [Spontaneous intracerebral hemorrhage: the clinical neuroradiological view]. *Radiologe* 1999; 39(10):828-837.

s15 McCarron MO, Nicoll JA, Love S, Ironside JW. Surgical intervention, biopsy and APOE genotype in cerebral amyloid angiopathy-related haemorrhage. *Br J Neurosurg* 1999; 13(5):462-467.

s16 Itoh Y, Yamada M. Cerebral amyloid angiopathy in the elderly: the clinicopathological features, pathogenesis, and risk factors. *Journal of Medical & Dental Sciences* 1997; 44(1):11-19.

s17 Iwamoto N, Ishihara T, Ito H, Uchino F. Morphological evaluation of amyloid-laden arteries in leptomeninges, cortices and subcortices in cerebral amyloid angiopathy with subcortical hemorrhage. *Acta Neuropathol* 1993; 86(5):418-421.

s18 Lange M, Feiden W. Amyloid angiopathy--a rare cause of intracerebral hemorrhage. *Neurosurg Rev* 1991; 14(4):297-301.

s19 Ishihara T, Takahashi M, Yokota T, Yamashita Y, Gondo T, Uchino F et al. The significance of cerebrovascular amyloid in the aetiology of superficial (lobar) cerebral haemorrhage and its incidence in the elderly population. *Journal of Pathology* 1991; 165(3):229-234.

s20 Okazaki H, Reagan TJ, Campbell RJ. Clinicopathologic studies of primary cerebral amyloid angiopathy. *Mayo Clinic Proceedings* 1979; 54(1):22-31.

s21 Greenberg SM, Finkelstein S, Schaefer P. Petechial hemorrhages accompanying lobar hemorrhage: detection by gradient echo MRI. *Neurology* 1996; 46:1751-1754.

s22 Itoh Y, Yamada M, Hayakawa M, Otomo E, Miyatake T. Cerebral amyloid angiopathy: a significant cause of cerebellar as well as lobar cerebral hemorrhage in the elderly. *Journal of the Neurological Sciences* 1993; 116(2):135-141.

s23 Dye JA, Rees G, Yang I, Vespa PM, Martin NA, Vinters HV. Neuropathologic analysis of hematomas evacuated from patients with spontaneous intracerebral hemorrhage. *Neuropathology* 2014; 34(3):253-260.

s24 Li XQ, Su DF, Chen HS, Fang Q. Clinical Neuropathological Analysis of 10 Cases of Cerebral Amyloid Angiopathy-Related Cerebral Lobar Hemorrhage. *J Korean Neurosurg Soc* 2015; 58(1):30-35.

s25 Knudsen KA, Rosand J, Karluk D, Greenberg SM. Clinical diagnosis of cerebral amyloid angiopathy: validation of the Boston criteria. *Neurology* 2001; 56(4):537-539.

s26 Charidimou A, Jaunmuktane Z, Baron JC, Burnell M, Varlet P, Peeters A et al. White matter perivascular spaces: An MRI marker in pathology-proven cerebral amyloid angiopathy? *Neurology* 2013.

s27 Ruano L, Samoes R, Taipa R, Melo Pires M. The aetiology of spontaneous intracerebral haemorrhage: Insights from a neuropathological series. Journal of Neurology . 2014.

Ref Type: Abstract

s28 Du Plessis DG, Dawson T, Whitwell H. Acute hemodynamic stress-another risk factor for cerebral amyloid angiopathy related intracerebral hemorrhage? Clinical Neuropathology . 2012.

Ref Type: Abstract

s29 Martinez-Ramirez S, Romero JR, Gurol ME, Ashkan S, McKee AC, Van Etten E et al. Diagnostic value of lobar hemorrhages for cerebral amyloid angiopathy in hospital and community-based individuals: A pathological correlation study. Stroke . 2014.

Ref Type: Abstract

s30 Suzuki K, Yanagawa T, Yoshikawa S, Ooigawa H, Takeda R, Nakajima H et al. Lobar cerebral hemorrhage due to amyloid angiopathy: Correlation between pathological evidence and outcome. Cerebrovasc Dis . 2013.

Ref Type: Abstract

s31 Li Y, Maeda M, Kida H, Ito A, Shindo A, Taniguchi A et al. Cortical microinfarcts as a putative MRI marker for cerebral amyloid angiopathy. Stroke . 2013.

Ref Type: Abstract

s32 Duodu Y, Mathews S, El Tawil S. Cerebral amyloid angiopathy (CAA), an often missed cause of intracranial bleeding. Journal of the Neurological Sciences . 2013.

Ref Type: Abstract

s33 Takeda S, Yamazaki K, Ikuta F, Arai H. Cerebral amyloid angiopathy related hemorrhage (CAA-H) occurs in the cerebral sulcus: Examination of a case, in which the initial hemorrhage occurred in the central sulcus. Neuropathology . 2012.

Ref Type: Abstract

s34 Lluch V, Lainez JM, Morera J, Cano M, Escudero J, Sancho J. [Spontaneous non-hypertensive cerebral hematomas]. *Arch Neurobiol (Madr )* 1989; 52(5):243-249.

s35 Ishii N, Nishihara Y, Horie A. [Lobar cerebral hemorrhage and amyloid angiopathy--a report of 4 autopsy cases]. *No To Shinkei* 1983; 35(2):167-174.

s36 Kuramatsu JB, Sauer R, Mauer C, Lücking H, Kloska SP, Kiphuth IC et al. Correlation of age and haematoma volume in patients with spontaneous lobar intracerebral haemorrhage. *Journal of Neurology, Neurosurgery & Psychiatry* 2011; 82(2):144-149.

s37 Maia LF, Vasconcelos C, Seixas S, Magalhaes R, Correia M. Lobar brain hemorrhages and white matter changes: Clinical, radiological and laboratorial profiles. *Cerebrovasc Dis* 2006; 22(2-3):155-161.

s38 Charidimou A, Meegahage R, Fox Z, Peeters A, Vandermeeren Y, Laloux P et al. Enlarged perivascular spaces as a marker of underlying arteriopathy in intracerebral haemorrhage: a multicentre MRI cohort study. *J Neurol Neurosurg Psychiatry* 2013; 84(6):624-629.

s39 Labro H, Al-Kadhimi Z, Djmil M, Oghlakian R, Alshekhlee A. Brain amyloidoma with cerebral hemorrhage. *J Am Osteopath Assoc* 2009; 109(7):372-375.

s40 Wong YK, Wu JJ, Hsu CC, Liao SY, Chung MT, Lee WH. Intracerebral hemorrhage caused by cerebral amyloid angiopathy: a case report. *Zhonghua Yi Xue Za Zhi (Taipei)* 1999; 62(1):55-60.

s41 Hosoi Y, Uchiyama T, Yoshida M, Takechi D, Shimizu T, Ohashi T et al. [A case of cerebral amyloid angiopathy with reversible white matter lesions and multiple cerebral microbleeds]. *Rinsho Shinkeigaku* 2012; 52(2):90-95.

s42 Tang YJ, Zhao JZ, Li Y, Sun YL. [The study of association between spontaneous intracranial hemorrhage and cerebral amyloid angiopathy]. *Zhonghua Yi Xue Za Zhi* 2010; 90(15):1016-1019.

s43 Tang YJ, Wang S, Zhu MW, Sun YL, Zhao JZ. Severe pathological manifestation of cerebral amyloid angiopathy correlates with poor outcome from cerebral amyloid angiopathy related intracranial hemorrhage. *Chin Med J (Engl )* 2013; 126(4):603-608.

s44 Li XQ, Han YL, Chen HS. Clinical and pathological study on 10 cases of cerebral lobe hemorrhage related with cerebral amyloid angiopathy. *Medical Journal of Chinese People's Liberation Army* 2015; 40(7):530-534.

s45 Mehndiratta P, Manjila S, Ostergard T, Eisele S, Cohen ML, Sila C et al. Cerebral amyloid angiopathy-associated intracerebral hemorrhage: pathology and management. *Neurosurg Focus* 2012; 32(4):E7.
