# Supplementary figures and images for: Imaging features of intracerebral hemorrhage with cerebral amyloid angiopathy: Systematic review and meta-analysis
Source: PLoS One. 2017 Jul 10;12(7):e0180923. doi: 10.1371/journal.pone.0180923 (PMC5507310; doi:10.1371/journal.pone.0180923)

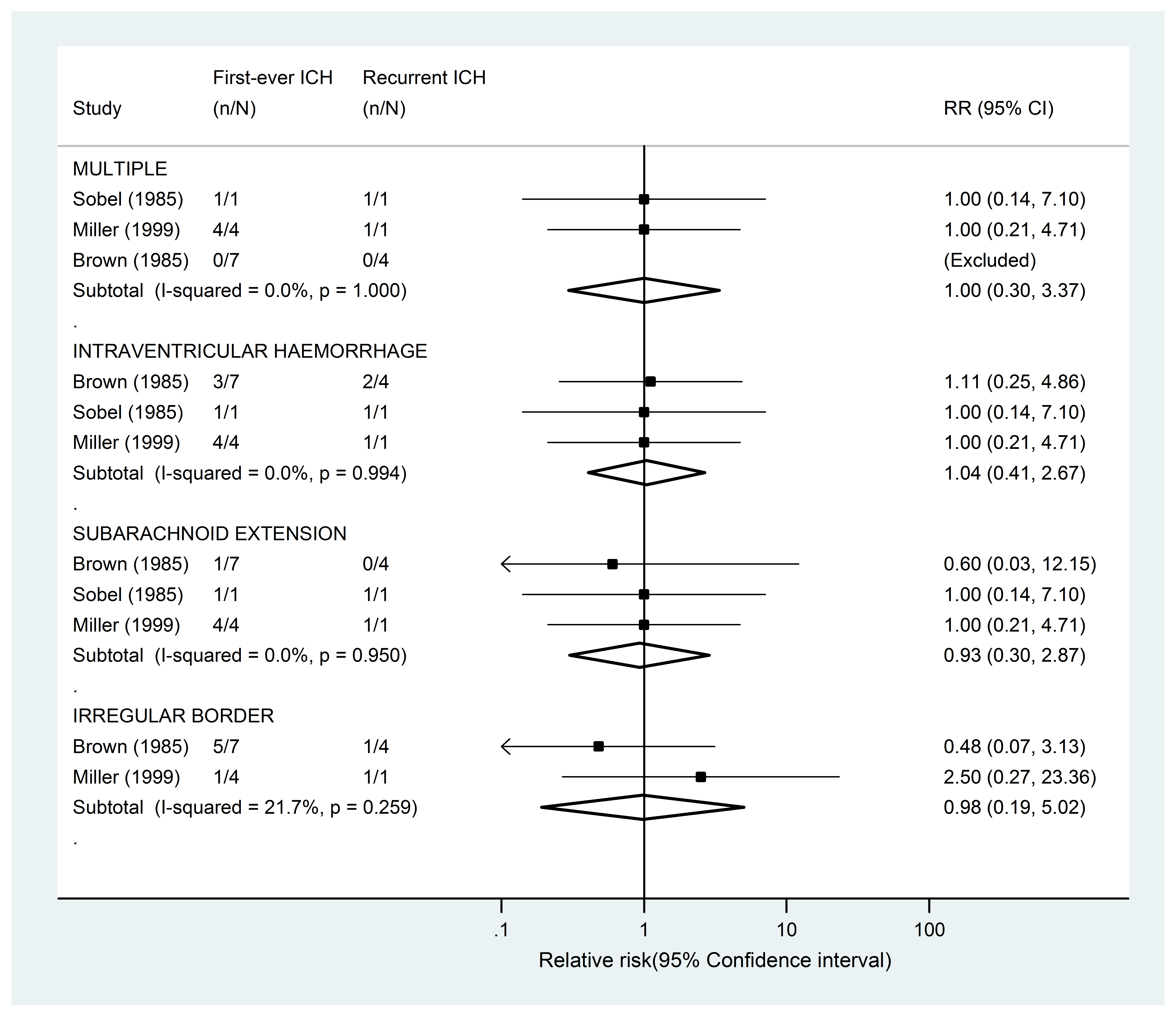

Supplement: S1 Fig — n = number of participants with feature, N = denominator, RR = relative risk, (95% CI) = 95% confidence intervals. (TIF) [file pone.0180923.s003.tif]

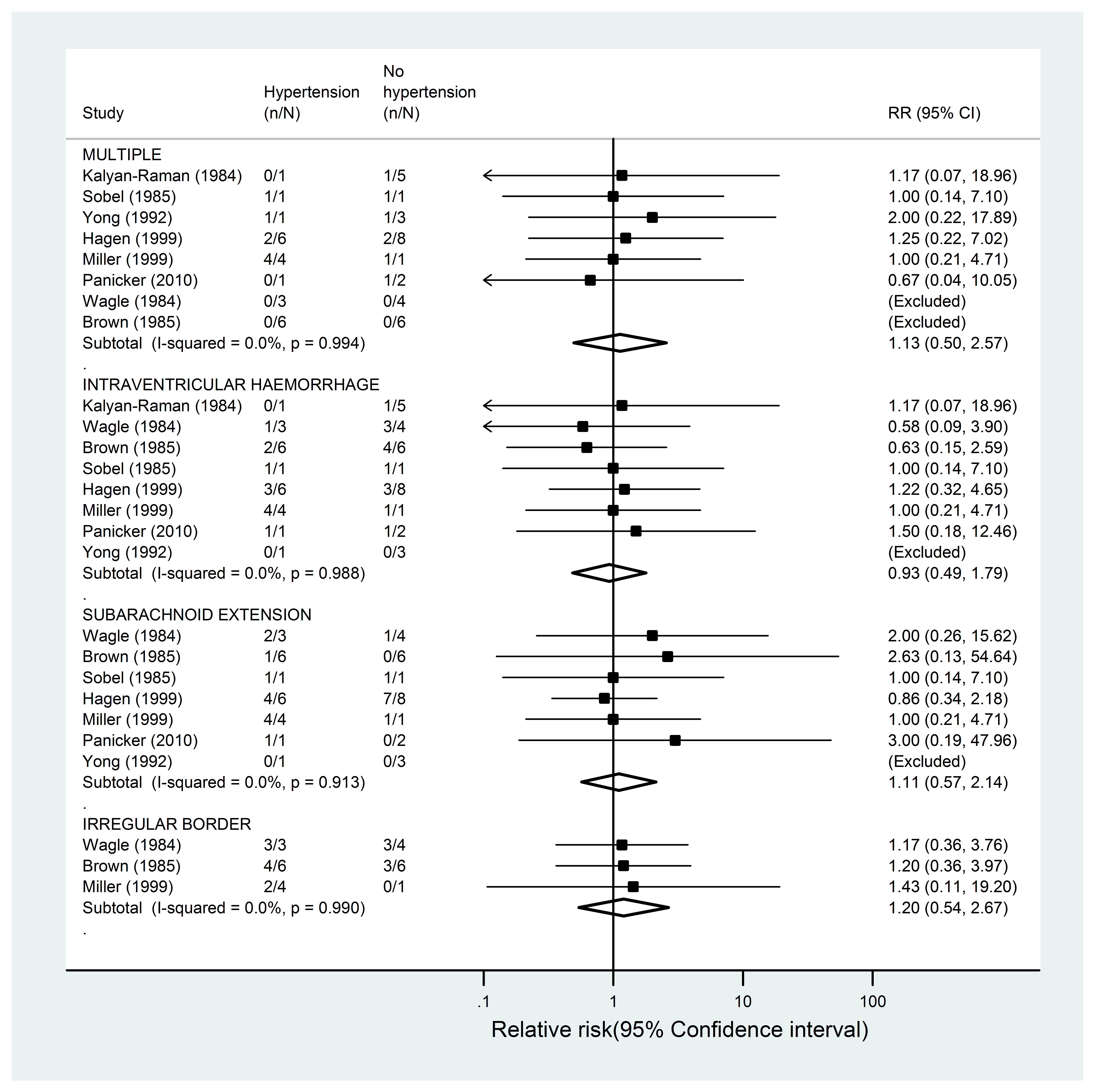

Supplement: S2 Fig — n = number of participants with feature, N = denominator, RR = relative risk, (95% CI) = 95% confidence intervals. (TIF) [file pone.0180923.s004.tif]

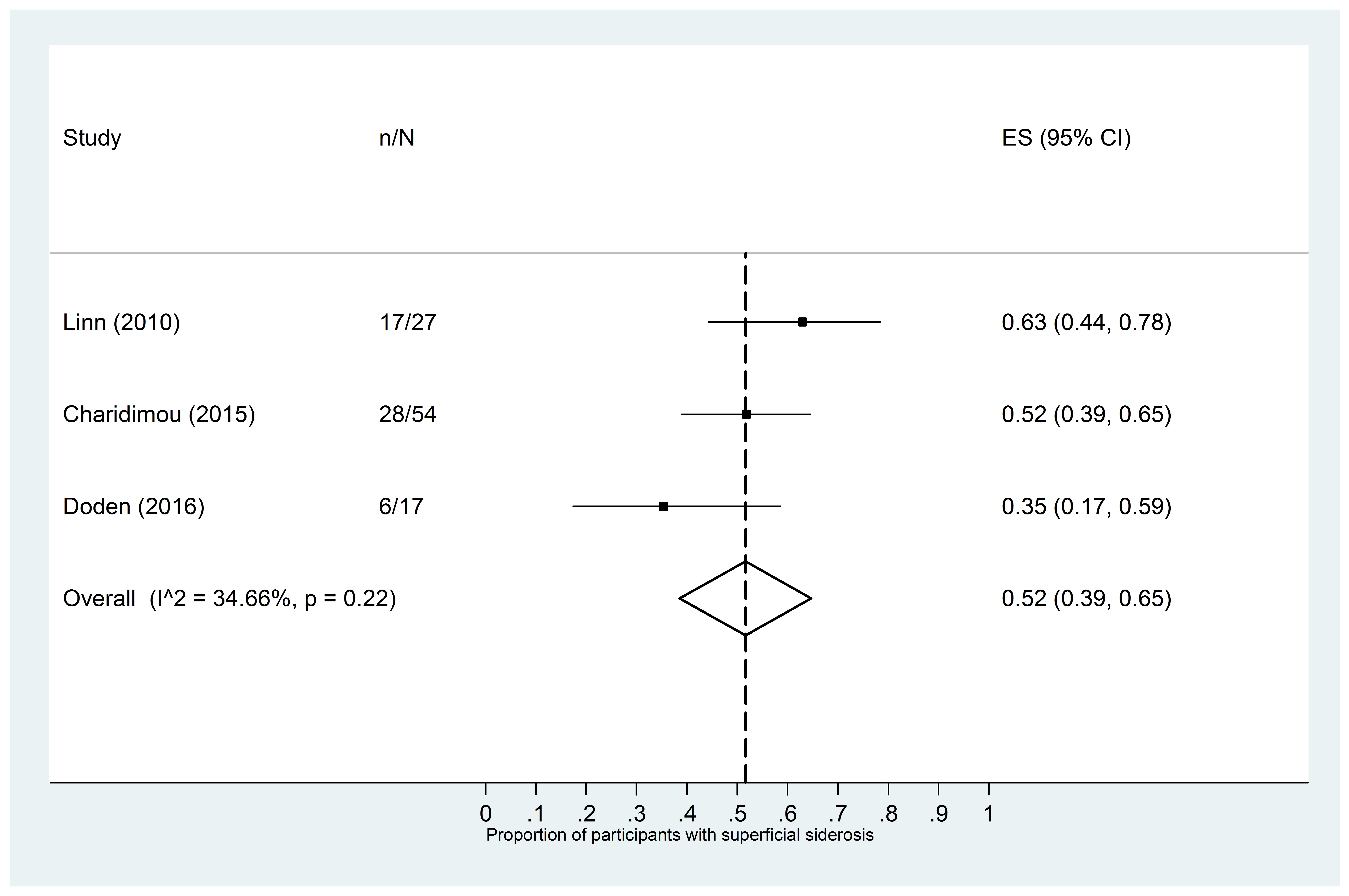

Supplement: S3 Fig — ES = effect size, n = number of participants with superficial siderosis, N = total number with CAA-associated ICH, (95% CI) = 95% confidence intervals. (TIF) [file pone.0180923.s005.tif]
